# Effect of Gating Modifier Toxins on Membrane Thickness: Implications for Toxin Effect on Gramicidin and Mechanosensitive Channels

**DOI:** 10.3390/toxins5020456

**Published:** 2013-02-22

**Authors:** Rong Chen, Shin-Ho Chung

**Affiliations:** Research School of Biology, Australian National University, Canberra, ACT 0200, Australia; E-Mail: shin-ho.chung@anu.edu.au

**Keywords:** membrane, lipid bilayer, gating modifier toxin, molecular dynamics

## Abstract

Various gating modifier toxins partition into membranes and interfere with the gating mechanisms of biological ion channels. For example, GsMTx4 potentiates gramicidin and several bacterial mechanosensitive channels whose gating kinetics are sensitive to mechanical properties of the membrane, whereas binding of HpTx2 shifts the voltage-activity curve of the voltage-gated potassium channel Kv4.2 to the right. The detailed process by which the toxin partitions into membranes has been difficult to probe using molecular dynamics due to the limited time scale accessible. Here we develop a protocol that allows the spontaneous assembly of a polypeptide toxin into membranes in atomistic molecular dynamics simulations of tens of nanoseconds. The protocol is applied to GsMTx4 and HpTx2. Both toxins, released in water at the start of the simulation, spontaneously bind into the lipid bilayer within 50 ns, with their hydrophobic patch penetrated into the bilayer beyond the phosphate groups of the lipids. It is found that the bilayer is about 2 Å thinner upon the binding of a GsMTx4 monomer. Such a thinning effect of GsMTx4 on membranes may explain its potentiation effect on gramicidin and mechanosensitive channels.

## 1. Introduction

Venomous animals, such as arachnids [1,2,3,4,5], reptiles [6,7] and marine invertebrates [8,9,10,11], have a vast array of toxins, targeted to certain biological ion channels such as voltage-gated K^+^ and Na^+^ channels. These highly structured and compact mini-proteins have evolved over millions of years for rapid paralysis of prey. These toxins are known to act on ion channels in at least two different ways. Pore blocker toxins physically occlude the ion-conducting pathway by inserting the side chain of a basic residue into the selectivity filter, while forming one or more hydrogen bonds with the polar residues on the wall of the external vestibule. Some extensively studied examples of pore blockers are ShK [12] and conotoxins [13] which are isolated from the venoms of sea anemone and cone snails, respectively, and maurotoxin [14] and OSK1 [15] from scorpion venoms. Gating modifier toxins, on the other hand, often interfere with the gating properties of ion channels by binding to the voltage sensor. Among the well-characterized gating modifiers are spider toxins [5] and scorpion α- and β-toxins [16,17]. From the extracellular space, the linker loops of scorpion toxins wedge into the binding pocket in the voltage sensor of sodium channels [18,19]. Once bound, the toxin shifts the activation curve either to the right or left, or prolongs the open time of a sodium channel by preventing the inactivation process from taking place. 

There is a class of small, globular polypeptide toxins extracted from spider venoms which partition into the membrane. Some among these are Scodra griseipes toxin 1 (SGTx1) [20], hanatoxin 1 (HaTx1) [21], voltage sensor toxin 1 (VSTx1) [22] and heteropodatoxin 2 (HpTx2) [23]. HaTx1 and SGTx1 are antagonists for *Shaw*-type K^+^ (Kv2) channels [21,24], whereas HpTx2 is a selective inhibitor for *Shal*-type K^+^ (Kv4) channels [25]. VSTx1, on the other hand, selectively inhibits the archeabacterial K^+^ channel KvAP [22,26]. The membrane partitioning of these toxins has been examined experimentally using fluorescence titration techniques [22,27,28,29], which suggest that HaTx1 and SGTx1 partition into membranes, especially those containing anionic lipids at neutral pH. On the other hand, HpTx2 partitions into membranes only in acidic conditions where the side chains of its acidic residues are protonated. After partitioning into the membrane, the toxins are believed to interact with the voltage sensors of ion channels buried in the lipids. 

Of special interest is a 34-residue toxin, GsMTx4, isolated from the tarantula *Grammostola spatulata* [30], which bears structural similarity with the other toxins penetrating into the membrane. The toxin carries an overall charge of +5*e* at neutral pH and a segment predominantly consisting of hydrophobic residues. This hydrophobic surface is surrounded by a ring of polar residues. GsMTx4 selectively inhibits stretch-activated cationic channels [30] and increases the opening rate and prolongs the mean open time of several bacterial mechanosensitive channels [31] and gramicidin channels [32]. In contrast to other gating modifier toxins, GsMTx4 is believed to modify the gating properties of the gramicidin and mechanosensitive channels, not by directly interacting with the proteins, but indirectly, by interfering with the local packing of the lipids surrounding the channels [32]. 

Several molecular dynamics (MD) simulation studies have been carried out to understand the binding mechanism of gating modifier toxins in model membranes [33,34,35,36,37,38]. In these studies, the toxins are frequently embedded into the lipid bilayer at different depths at the start of the simulations to probe the most preferred position of toxin binding. Such an approach suffers from several limitations. First, as there are numerous possible ways of placing a toxin initially, one has to make an arbitrary decision on the initial configuration of the toxin relative to the membrane. Second, simulations started from different positions or orientations of the toxin do not always lead to a unique binding mode. As the system often fails to evolve to the global minimum energy state on a limited time scale, the final state tends to be highly dependent on the initial position of the toxin [38]. Third, this approach is not efficient if it is applied to a toxin that binds to the membrane weakly. In the case of a weakly binding toxin, the prior knowledge introduced in generating the starting structures may significantly bias the simulation outcome. Ideally, the toxin should be released in water and allowed to bind to the membrane spontaneously, such that the simulation reproduces the binding process realistically. Such a simulation would provide strong evidence on whether or not a toxin penetrates into the membrane, an important property for understanding the mechanism of action by the toxin. Unfortunately, the aggregation of peptides into planar membranes requires a time scale beyond what is achievable for atomistic MD simulations. For example, Chen [39] showed that a 29-residue globular peptide, known as kalata B1, aggregates into the lipid bilayer only once in six simulations, each simulation lasting more than 200 ns [39]. The initial binding of kalata B1 to the bilayer surface is fast, but the penetration of the peptide into the hydrophobic core of the bilayer is much slower [39]. To achieve the required computational efficiency, the amount of detail in the system is often reduced by using coarse-grained models [40]. Alternatively, an efficient sampling protocol such as the generalized shadow hybrid Monte Carlo method [41] can be used. 

To circumvent these shortcomings, we introduce here a novel methodology that can examine how polypeptide toxins and possibly other membrane-active peptides penetrate into membranes using atomistic MD simulations of tens of nanoseconds. This method takes advantage of the fact that various membrane-active peptides bind favorably to membrane regions with positive curvature [39,42]. The simulation protocol is described in detail in the Methods section. We apply this protocol to two gating modifier toxins, GsMTx4 and HpTx2. We show that both GsMTx4 and HpTx2 partition into membranes, but HpTx2 binds much less strongly. We find that the bilayer is significantly thinner upon GsMTx4 binding. This finding has broad implications for understanding the effect of GsMTx4 on the gating kinetics of mechanosensitive [31] and gramicidin [32] channels.

## 2. Results and Discussion

### 2.1. Binding of GsMTx4

**Figure 1:**
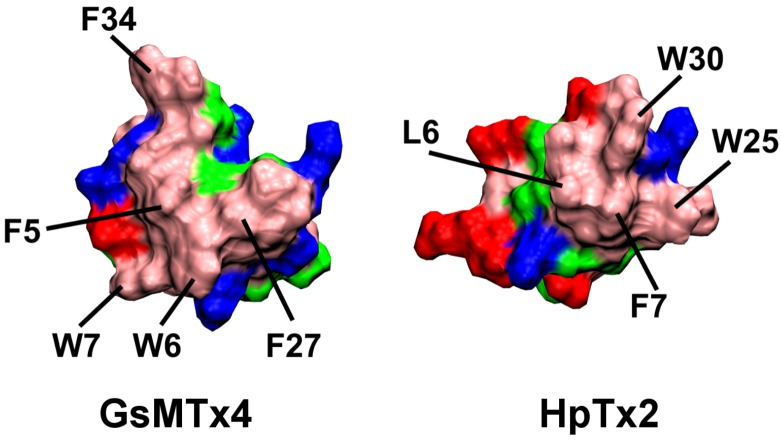
The molecular surface of GsMTx4 and HpTx2. Key hydrophobic residues are highlighted. Residues are colored as: basic, blue; acidic, red; polar, green; hydrophobic, pink.

First, we look at the toxin GsMTx4, which binds strongly to POPC bilayers according to the experiments of Posokhov *et al.* [28]. The amino acid sequence of GsMTx4 is GCLEF-WWKCN-PNDDK-CCRPK-LKCSK-LFKLC-NFSF. The *C*-terminus of the toxin is amidated. The hydrophobic patch of GsMTx4 containing residues Trp^6,7^ and Phe^5,27,34^ is displayed in Figure 1. Also shown in the figure is the hydrophobic patch of HpTx2, which will be discussed later. To investigate the binding of GsMTx4 to POPC bilayers, we place the toxin near a water-filled pore in the middle of the bilayer, as described in the Methods section. This hydrophilic pore is created to facilitate the insertion of the toxin into the bilayer. Three simulations starting from different initial random velocities, which we will refer to as GT-sim1, GT-sim2 and GT-sim3, are performed.

**Figure 2:**
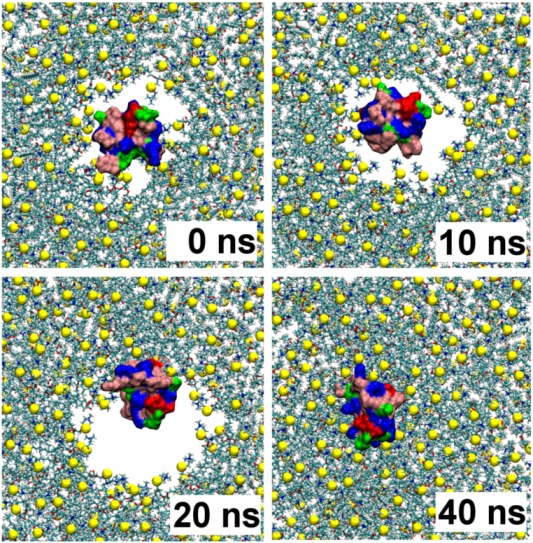
The position of GsMTx4 relative to the bilayer viewed along the bilayer normal over the first 40 ns of the simulation GT-sim1. Color representation of toxin is the same as Figure 1.

**Figure 3:**
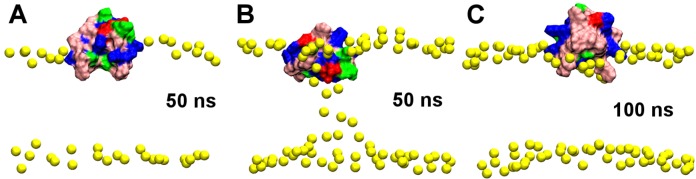
GsMTx4 bound to a POPC bilayer from simulations GT-sim1 at 50 ns (**A**) and GT-sim2 at 50 ns (**B**) and 100 ns (**C**). Yellow spheres represent phosphorus atoms of lipids. Color representation of toxin is the same as Figure 1.

In the simulation GT-sim1, the toxin is observed to penetrate deeply into the bilayer during the first 20 ns, after which the toxin remains bound and the pore progressively disappears, as illustrated in Figure 2. After GsMTx4 enters the water-filled pore of the bilayer, the toxin quickly attaches itself to the polar heads located at its edge (Figure 2, 20 ns), and, subsequently, the pore collapses and the polar heads tightly enwrap the toxin (Figure 2, 40 ns). The pore is wide enough such that the toxin is observed to tumble freely within the pore. The hydrophobic patch of the toxin penetrates beyond the head groups of lipids at 50 ns, while a ring of polar residues around this patch are located at the interface between the lipid head groups and water (Figure 3A). This hydrophobic patch corresponds closely to the key hydrophobic residues of toxin SGTx1 determined experimentally [27]. The center of the deepest penetrating residue, Phe^27^, is 14–15 Å from the bilayer center. The simulation is continued until 80 ns to ensure that the system is fully equilibrated, and the toxin-bilayer complex is found to be stable. At the end of the simulation, the leaflet to which GsMTx4 bound is smaller by six lipids compared to the opposite leaflet. The surface area of six lipids (≈380 Å^2^) is comparable to the size of the hydrophobic patch of GsMTx4. 

In the simulation GT-sim2, GsMTx4 is observed to interact with the lipids from the leaflet opposite to which it binds using its polar segments (Figure 3B). However, the polar segments gradually move away from the core of the bilayer to the water phase and two phenylalanine residues of its hydrophobic patch penetrate into the bilayer when the simulation is extended to 100 ns (Figure 3C), leading to a binding mode similar to that shown in Figure 3A. In the binding mode of Figure 3C, Phe^27,34^ are buried near the hydrophobic core of the bilayer, while polar residues interact with lipid head groups or water, suggesting that the binding mode of Figure 3B is less stable than that of Figure 3A. The two leaflets of the bilayer are found to contain an equal number of lipids after redistribution of lipids during pore collapse. 

In the simulation GT-sim3, the same binding mode as that shown in Figure 3A is obtained. In this case, the two leaflets of the bilayer contain 126 and 130 lipids, respectively. We repeat the simulation three additional times with a different position and orientation of the toxin, and a similar pattern of binding is observed. Thus, the initial position of the toxin has no effect on the simulation outcome.

The simulations of GsMTx4 described above demonstrate that GsMTx4 binds most favorably to the interfacial region of the lipid bilayer. Nishizawa and Nishizawa [38] found that two binding modes, referred to as shallow mode and deep mode, are possible for the binding of GsMTx4 to membranes. In the shallow mode, the hydrophobic patch of the toxin penetrates into the bilayer but the polar residues of the toxin remain in the water phase or interact with lipid head groups [38]. In the deep mode, some of the polar residues of the toxin are located near the center of the bilayer and interact with the head groups of the lipids from the opposite leaflet [38]. It was proposed that the two binding modes for the binding of the toxin to POPC bilayers are equally favorable [38]. Our simulations are consistent with the findings of Nishizawa and Nishizawa [38]. For example, the binding mode of Figure 3A is similar to the shallow mode of Nishizawa and Nishizawa [38], whereas the binding mode of Figure 3B is comparable to their deep mode. However, our simulations suggest that the shallow mode is more favorable than the deep mode, as the deep mode evolves spontaneously to the shallow mode over a simulation period of 50 ns (Figure 3B,C). The interfacial binding by GsMTx4 observed here is consistent with the binding modes of SGTx1 [34] and VSTx1 [35] to POPC bilayers observed in previous computational studies.

### 2.2. Binding of HpTx2

**Figure 4:**
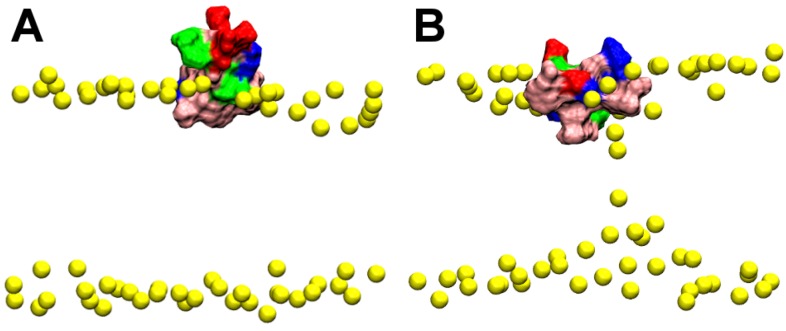
HpTx2 bound to a POPC bilayer at 50 ns of the simulations HT-sim1 (**A**) and HT-sim2 (**B**). Yellow spheres represent phosphorus atoms of lipids. Color representation of toxin is the same as Figure 1.

Next we look at the toxin HpTx2, which binds weakly to POPC bilayers at neutral pH [28]. The primary structure of HpTx2 is DDCGK-LFSGC-DTNAD-CCEGY-VCRLW-CKLDW. The key hydrophobic residues of the toxin are shown in Figure 1. Two simulations are performed, referred to as HT-sim1 and HT-sim2. In the simulation HT-sim1, the pore collapses completely after 50 ns; the toxin penetrates into the bilayer with the center of Trp^25^ being ≈15 Å from the bilayer center (Figure 4A). The two leaflets of the bilayer contain the same number of lipids after pore collapse. The simulation is continued until 80 ns, and the position of the toxin relative to the bilayer remains unchanged. In the simulation HT-sim2, the pore does not collapse completely after 50 ns (Figure 4B), but the binding mode is consistent with that observed in the simulation HT-sim1. In both simulations HT-sim1 and HT-sim2, the hydrophobic patch, primarily consisting of four residues Leu^6^, Phe^7^ and Trp^25,30^, penetrates into the bilayer beyond the phosphate groups of lipids. To examine the effect of lipid distribution on toxin binding, we extend the simulation HT-sim1 at 50 ns for a further 40 ns with four lipids of the toxin-bound leaflet removed. No significant changes in the position of the toxin are observed, suggesting that lipid distribution has minimal effect on toxin binding. The three simulations of HpTx2 demonstrate that HpTx2 also partitions into the interfacial region of lipid bilayers, similar to GsMTx4. 

### 2.3. Energetics of Toxin Binding

The results of our simulations reveal that both GsMTx4 and HpTx2 bind to the interfacial region of POPC bilayers. However, experimental measurements suggest that GsMTx4 but not HpTx2 binds POPC bilayers strongly [28]. To reconcile this discrepancy, we quantify the strength of the toxin binding by deriving the one-dimensional PMF profile. The profiles constructed along the bilayer normal show that the binding of HpTx2 to the lipid bilayer is weaker than that of GsMTx4. This observation is in accord with experiment. 

**Figure 5:**
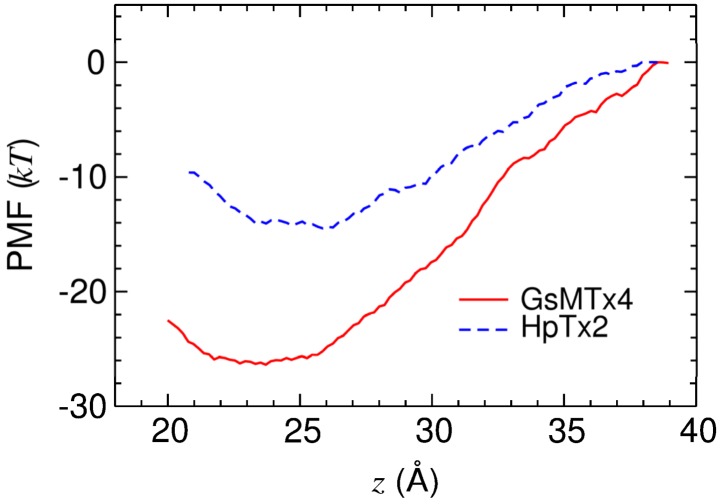
The PMF profiles for the binding of GsMTx4 and HpTx2 to POPC bilayers. The reaction coordinate, *z*, is the distance between the centers of mass of the toxin and the phosphorus atoms of lipids along the bilayer normal. The random errors of both profiles are approximately 0.3 kT.

The PMF profiles for the binding of GsMTx4 and HpTx2 to POPC bilayers are displayed in Figure 5. The depths of the profiles are 15 kT for HpTx2 and *26* kT for GsMTx4, thus demonstrating that the latter binds to the bilayer more strongly than the former. We note here that the depth of the PMF profile for GsMTx4 predicted from experiment is approximately 12 kT [28], or about half of that we derive computationally. Similar discrepancies between the energy values derived from computational methods and experiment have been reported previously. For example, the well depth of the PMF profile for VSTx1 was calculated to be 38–39 kT [36], compared to the value of 12 kT expected from experiment [22]. Such large discrepancies may result from a systematic error in the calculated PMF profile due to incomplete sampling of all possible toxin orientations in the umbrella sampling simulations. The PMF profile of even small molecules partitioning into membranes converges rather slowly on a time scale of hundreds of nanoseconds, as demonstrated by Neale *et al*. [43]. It is also possible that the free energy values derived computationally are not directly comparable to the experiment. For example, toxin peptides in solution could assemble through their hydrophobic residues into multimers, reducing the concentration of the toxin that can bind the membrane effectively. Therefore, the binding free energy measured experimentally could also contain a systematic error. Nevertheless, the higher binding affinity of GsMTx4 relative to HpTx2 predicted from the PMF calculations is in accord with experiment [28].

As shown in Figure 5, the interfacial region 22–25 Å above the center of the bilayer provides the most favorable site for the binding of the two toxins we examined (see also Figure 3 and Figure 4). This conclusion is consistent with previous computational and experimental studies. For example, SGTx1 also binds most favorably to this interfacial region [34]. The position of the PMF minima observed here is similar to that for the binding of VSTx1 to several different types of lipid bilayers, including POPC determined by Wee *et al.* [35,36]. Moreover, HaTx1 binds to the interfacial region of lipid bilayers according to the experimental measurements of Phillips *et al.* [29]. Therefore, the interfacial region of lipid bilayers may form a common binding site for gating modifier toxins.

### 2.4. Bilayer Thickness

Having demonstrated the binding modes of GsMTx4 and HpTx2 into POPC bilayers, we examine the effect of toxin binding on membrane properties, specifically, hydrophobic thickness (*d*_H_). The *d*_H_ of the lipid bilayer is estimated as the difference between the average *z* coordinates of the second carbon atoms of lipid acyl chains in each leaflet [44]. The last 10 ns of each simulation are used for data analysis. It is found that the bilayer becomes significantly thinner upon the binding of the toxins, especially GsMTx4.

The average *d*_H_ values are 26.3 and 26.0 Å, respectively, over the last 10 ns of the simulations GT-sim1 and GT-sim3 for GsMTx4, in which the hydrophobic patch of the toxin is fully buried in the bilayer. In the simulation GT-sim1, no systematic drift in the d_H_ values is observed over the last 20 ns, indicating that the bilayer is well equilibrated. These values are significantly lower than the value of 28.3 Å for a control simulation (referred to as PC-sim2) in which no toxin is present. When the final configuration of GT-sim3 is further simulated for 40 ns after the toxin is removed, the thickness of the bilayer increases from 26.0 to 27.3 Å. Although the bilayer is not expected to equilibrate fully in 40 ns, the fact that the bilayer thickness increases substantially after the removal of GsMTx4 demonstrates that the binding of GsMTx4 has a direct effect on bilayer thickness. To examine the change in bilayer thickness as a function of toxin concentration, simulation GT-sim4 is started from GT-sim1 with one more GsMTx4 peptide added into the system. In the starting configuration of the simulation GT-sim4, nine lipids overlapping with the second toxin peptide are removed. The d_H_ value is found to be 25.5 Å over the last 10 ns of the simulation GT-sim4. In the simulation GT-sim2, the bilayer is slightly thicker (*d*_H_ = 27.2 Å) than that in GT-sim1 and GT-sim3, possibly because the hydrophobic patch of the toxin is not fully buried in the bilayer and the bilayer is symmetrical. This series of simulations demonstrate the thinning effect of GsMTx4 on bilayer thickness.

**Table 1:** The hydrophobic thickness of bilayer (*d*_H_) as a function of the distance between the toxin and the lipids in the bilayer plane. Simulation GT-sim1 is used for data analysis. Standard deviations are given (*n* = 500).

Distance from toxin center (Å)	No. of lipids in one leaflet	*d*_H_ (Å)
<15	8.3 ± 2.6	27.9 ± 1.6
15–25	16.1 ± 3.7	27.2 ± 1.0
25–35	29.4 ± 4.9	26.0 ± 0.5
>35	71.2 ± 4.1	25.8 ± 0.5
Total	125	26.3 ± 0.2

To measure the dimpling effect of GsMTx4 on the bilayer, we calculate *d*_H_ as a function of the distance between the toxin and lipids. The results tabulated in Table 1 reveal that the *d*_H_ value of the lipids that are in direct contact with the toxin (distance less than 15 Å) is comparable to the *d*_H_ value of the bilayer with no toxin (27.9 *vs.* 28.3 Å). The bilayer thickness in the regions beyond 25 Å from the center of mass of the toxin is 2 Å, or 7%, less than the pure bilayer. Therefore, GsMTx4 perturbs the bilayer thickness through a long-range effect. This is in contrast to some other membrane peptides such as voltage sensors of potassium channels [45] and gramicidin channels [46], which are fully embedded in the membrane rather than bound to the surface. These peptides perturb lipids in close proximity to them and have a minimal long-range effect [45,46]. Considering that partial charges of atoms in the lipid head group can have a drastic effect on bilayer thickness and area [47], GsMTx4, which carries an overall charge of +5*e*, may induce a long-range thinning effect on the bilayer mainly through electrostatic interactions with lipid head groups. 

In the simulation HT-sim1 of HpTx2, in which the pore in the bilayer collapses completely, the *d*_H_ value is, on average, 27.6 Å, which is slightly lower than that of the pure POPC bilayer of 128 lipids/leaflet in the control simulation PC-sim2 without the toxin (*d*_H_ = 28.3 Å). In the simulation HT-sim3, in which the bilayer contains 124 and 128 lipids in the two leaflets, the *d*_H_ value is found to be 26.8 Å. Note that in HT-sim1 both leaflets have 128 lipids. A comparison of the *d*_H_ values determined from simulations HT-sim1 and HT-sim3 suggests that redistribution of lipids can also remodel bilayer thickness.

The analysis of bilayer thickness described above show that both GsMTx4 and HpTx2 have a profound effect on the thickness of the bilayer. The thinning effect of the toxins on the bilayer is due to toxin binding and the redistribution of lipids in the two leaflets.

### 2.5. Implications for Toxin Effect on Ion Channels

The effect of GsMTx4 on the thickness of the lipid bilayer observed here can be linked to the potentiation effect of the toxin on gramicidin [32] and bacterial mechanosensitive channels MscS and MscL [31,48] observed experimentally. The lifetime of gramicidin doubles after the addition of 200 nM GsMTx4 [32], and the addition of 10 μM GsMTx4 reduces the membrane tension required to open MscS and MscL [31]. Both gramicidin [49] and MscL [50] have been shown to open at higher probabilities in thinner bilayers. For example, the mean channel life of gramicidin can be doubled by decreasing the hydrophobic thickness by 2.4 Å [49], a magnitude comparable to the thinning effect of one monomer GsMTx4 on the POPC bilayer of 128 lipids per leaflet observed here. In addition, direct interactions between GsMTx4 and gramicidin are not likely to be required for toxin action [32]. Therefore, consistent with previous views [31,51], our results suggest that GsMTx4 increases the mean open time of gramicidin channels and the opening rate of the bacterial MscS channel by inducing thin membranes.

GsMTx4 has also been shown to inhibit certain mechanosensitive channels [30,32] and interfere with the gating of MscS in a concentration-dependent manner [48]. These experimental observations might be due to the fact that the toxin can have a thickening or thinning effect, depending on the composition of the membrane. Further experimental and computational studies on how GsMTx4 inhibits these channels in different model membranes are warranted.

## 3. Methods

### 3.1. Simulation Protocol

**Figure 6:**
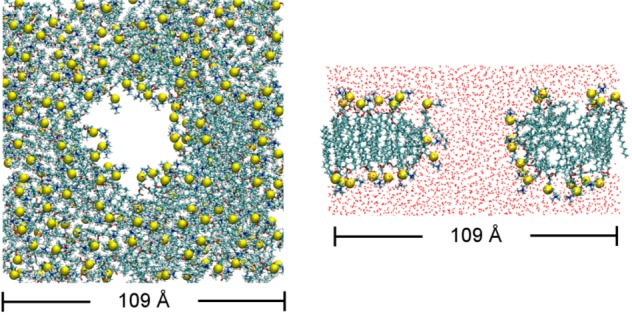
The POPC bilayer (128 lipids per leaflet) containing a water-filled pore after 20 ns of simulation under constant area, viewed along (left panel) and perpendicular to (right panel) the bilayer normal. Yellow spheres represent phosphorus atoms of lipids.

We first create a water-filled pore in a POPC (1-palmitoyl-2-oleoyl-*sn*-glycero-3-phosphocholine) bilayer by removing all the lipids within 20 Å of an arbitrary lipid in the bilayer center. The resulting bilayer consisting of 128 lipids per leaflet is solvated with 12,671 water molecules and 46 K^+^/Cl^−^ ions each, corresponding to a salt concentration of 0.2 M. The system is simulated under constant area for a period of 20 ns, allowing a water-filled toroidal pore to be formed in the bilayer. Such a pore, known as a lipid ion channel, occurs naturally in membranes [52,53]. During the simulation, labeled as PC-sim1, the lipid head groups bend toward the pore, creating a positive curvature (Figure 6). 

Subsequently, the toxin, GsMTx4 [54] or HpTx2 [55], is placed near the entrance of the pore. The protonation state of titratable groups within the toxin is chosen as appropriate for neutral pH 7. Each toxin system is simulated for 50–100 ns without restraints, except that the area of the bilayer is maintained constant during the first 20 ns. The toxin is observed to partition into the bilayer rapidly over the first 20 ns. Such a rapid partitioning of the toxin into the bilayer is in part facilitated by the positive curvature of the lipid head groups around the pore. It is unlikely that the pore is required by the membrane partitioning of the toxin. The area of the bilayer is allowed to evolve freely afterwards and the pore collapses rapidly. A summary of all the simulations performed is given in Table 2.

**Table 2:** A summary of the simulations performed.

Label	Toxin	Start	Time scale (ns)	Lipid distribution	*d*_H_ (Å) ^§^
PC-sim1 ^*^	-	-	20	128/128	-
PC-sim2		-	40	128/128	28.3 ± 0.3
PC-sim3		GT-sim3	40	126/130	27.3 ± 0.2
GT-sim1 ^†^	GsMTx4	PC-sim1	80	125/131	26.3 ± 0.2
GT-sim2 ^†^		PC-sim1	100	128/128	27.2 ± 0.3
GT-sim3 ^†^		PC-sim1	50	126/130	26.0 ± 0.4
GT-sim4 ^‡^		GT-sim1	30	116/131	25.5 ± 0.2
HT-sim1 ^†^	HpTx2	PC-sim1	80	128/128	27.6 ± 0.4
HT-sim2 ^†^		PC-sim1	50	-	-
HT-sim3		HT-sim1	40	124/128	26.8 ± 0.3

^*^ The bilayer contains a water-filled pore of ~20 Å in radius; ^†^ The area of the bilayer is maintained constant during the first 20 ns; ^‡^ Two GsMTx4 peptides are bound to one leaflet of the bilayer; ^§^ Standard deviations are given (*n* = 500).

Compared to the conventional method of placing the toxin at different depths in the bilayer at the start of the simulation, our protocol has several advantages. First, no prior knowledge is taken into account in constructing the starting structure of the simulation. The toxin can be placed at any depth in the pore in a random orientation. Second, only one starting structure needs to be constructed, as opposed to the multiple structures required by the conventional method. Third, our protocol can give good evidence on whether or not a toxin penetrates into the membrane. For example, HpTx2 does not have detectable binding for POPC bilayers at neutral pH [28], but we are able to demonstrate here that it actually can partition into the bilayer, albeit with low affinity.

### 3.2. Molecular Dynamics Simulations

MD simulations are performed at 1 atm and 300 K using NAMD 2.9 [56], with periodic boundary conditions applied. The CHARMM36 force field and the TIP3P model for water are used to describe the interatomic interactions in the systems [57,58,59]. The short-range non-bonded interactions are truncated at the switch and cutoff distances of 8.0 Å and 12.0 Å, respectively. The long-range electrostatic interactions are accounted for by the particle mesh Ewald method with a maximum grid spacing of 1.0 Å. The SHAKE [60] and SETTLE [61] algorithms are used to keep the bond lengths in the system rigid. A time step of 2 fs is used. Trajectories are saved every 20 ps for analysis. Molecular graphics are generated using VMD [62].

### 3.3. Umbrella Sampling

We derive the potential of mean force (PMF) profile for the toxin binding using the umbrella sampling method, which has been applied to similar systems in previous studies [35,36]. The distance between the center of mass (COM) of toxin backbone and the center of lipid phosphorus atoms along the bilayer normal (*z*) is chosen as the reaction coordinate. To generate the starting configuration of each umbrella window, a constant force is applied to pull the toxin away from its bound position along the bilayer normal. During the pulling, toxin backbone and the phosphorus atoms of lipids are maintained rigid using harmonic restraints. In subsequent umbrella sampling simulations, both toxin and lipids are free to move. Windows are spaced at 0.5 Å intervals. 

The COM of the toxin backbone is restrained to the center of each umbrella window along the bilayer normal using a harmonic force constant of 30 kcal/mol/Å^2^. Otherwise, the toxin is free to move. Each umbrella window is simulated for 5–8 ns until convergence of PMF is obtained. A convergence is assumed if the depth of PMF profile changes by 0.5 kT over the last 1 ns. The first 1 ns of each window, considered as equilibration, is removed from data analysis. The windows at *z* > 38.0 Å are assumed to be bulk, and the PMF of these windows is set to zero. In previous studies of VSTx1, the PMF was found to be zero at *z* > 36.0 Å [36]. The PMF profile is constructed using the weighted histogram analysis method [63]. The random error of the PMF profile is estimated using a bootstrapping method, as described previously [18].

## 4. Conclusions

We propose here an efficient sampling protocol for examining the assembly of membrane-active peptides such as gating modifier toxins into membranes using atomistic MD simulations. With this approach, there is no need to decide on the initial position and orientation of the toxin in the bilayer. The initial configuration evolves to the final stable state in simulations lasting several tens of nanoseconds. Using this novel methodology, we are able to predict the aggregation of two gating modifier toxins, GsMTx4 and HpTx2, into the interfacial region of a model membrane. Our findings are consistent with the experiment, as well as previous computational studies. For the first time, to the best of our knowledge, direct evidence on the thinning effect of GsMTx4 on membranes is obtained, which may explain the mechanism by which GsMTx4 potentiates gramicidin and bacterial MscS channels.
